# Test performance of faecal occult blood testing for the detection of bowel cancer in people with chronic kidney disease (DETECT) protocol

**DOI:** 10.1186/1471-2458-11-516

**Published:** 2011-06-29

**Authors:** Germaine Wong, Kirsten Howard, Jeremy R Chapman, Allison Tong, Michael J Bourke, Andrew Hayen, Petra Macaskill, Richard L Hope, Narelle Williams, Anh Kieu, Richard Allen, Steven Chadban, Carol Pollock, Angela Webster, Simon D Roger, Jonathan C Craig

**Affiliations:** 1 Sydney School of Public Health University of Sydney, Australia; 2 Centre for Transplant and Renal Research, Westmead Hospital, Australia; 3 Centre for kidney research, Kids Research Institute at the Children's Hospital at Westmead, Australia; 4 Department of Gastroenterology, Westmead Hospital, Australia; 5 Department of Gastroenterology, Blacktown Hospital, Australia; 6 Central Clinical School, Royal Prince Alfred Hospital, University of Sydney, Australia; 7 Kollings Institute, University of Sydney, Australia; 8 Department of Renal Medicine, Gosford Hospital, Australia

## Abstract

**Background:**

Cancer is a major cause of mortality and morbidity in patients with chronic kidney disease (CKD). In patients without kidney disease, screening is a major strategy for reducing the risk of cancer and improving the health outcomes for those who developed cancers by detecting treatable cancers at an early stage. Among those with CKD, the effectiveness, the efficacy and patients' preferences for cancer screening are unknown.

**Methods/Design:**

This work describes the protocol for the DETECT study examining the effectiveness, efficiency and patient's perspectives of colorectal cancer screening using immunochemical faecal occult blood testing (iFOBT) for people with CKD. The aims of the DETECT study are 1) to determine the test performance characteristics of iFOBT screening in individuals with CKD, 2) to estimate the incremental costs and health benefits of iFOBT screening in CKD compared to no screening and 3) to elicit patients' perspective for colorectal cancer screening in the CKD population. Three different study designs will be used to explore the uncertainties surrounding colorectal cancer screening in CKD. A diagnostic test accuracy study of iFOBT screening will be conducted across all stages of CKD in patients ages 35-70. Using individually collected direct healthcare costs and outcomes from the diagnostic test accuracy study, cost-utility and cost-effective analyses will be performed to estimate the costs and health benefits of iFOBT screening in CKD. Qualitative in-depth interviews will be undertaken in a subset of participants from the diagnostic test accuracy study to investigate the perspectives, experiences, attitudes and beliefs about colorectal cancer screening among individuals with CKD.

**Discussion:**

The DETECT study will target the three major unknowns about early cancer detection in CKD. Findings from our study will provide accurate and definitive estimates of screening efficacy and efficiency for colorectal cancer, and will allow better service planning and budgeting for early cancer detection in this at-risk population.

The DETECT study is also registered with the Australia New Zealand Clinical Trials Registry ACTRN12611000538943

## Background

After cardiovascular disease, the second most common cause of death in people with CKD is cancer(1,2). CKD both increases the risk of cancer and the likelihood of a poor prognosis. A two to three-fold increase in the overall cancer incidence had been reported in the dialysis and transplant populations(3), and in people with moderate CKD (stages III and IV). The development of cancer in people with CKD is associated with an excess risk of death of about 20-30% compared with those with cancer with normal kidney function. The increased risk of cancer in the transplant population is largely attributed to the effects of immunosuppression use. Among those with moderate CKD and on dialysis, the biological rationale for the increased risk remains unclear. Some have proposed the increased risk of cancer development may be due to the effects of chronic inflammation and uraemia, but there is a lack of definitive epidemiological and biological evidence to show a direct casual relationship(4,5).

### Colorectal cancer and CKD

Colorectal cancer is the second most common cause of solid organ cancer in recipients of kidney transplants and those on dialysis, with an excess risk of at least 2.5 and 1.4 times that of the age and gender matched general population(3). For colorectal cancer, the increase in risk is greatest for younger patients (ages 35-40) with a standard incidence ratio (SIR) of 13.5, and a SIR of 2.3 among older individuals (ages > 55) compared to the age and gender matched general population(6). Survival of these patients is also poor. One-year survival after colorectal cancer diagnosis is less than 50% for patients with a kidney transplant, and less than 30% for dialysis patients, with only 10% of dialysis patients survival 5 years after initial diagnoses(2).

### Screening for colorectal cancer in the general population

There is strong evidence that screening for colorectal cancer using FOBT is effective and reduces cancer-specific mortality by at least 16-33%(7). Population screening for colorectal cancer is recommended and applied in the general population worldwide. In England, the National Health Service (NHS) bowel cancer screening program was developed in 2006 and phased in over a period of 3 years for people aged 60-69. Similar programs were also developed in Scotland, Wales and Northern Ireland, with a recent extension of the Welch and English program extending to men and women aged 70 - 74(8-10). In Australia, iFOBT was first offered to those aged 50, 55 and 65 as part of the initial roll-out of the National Bowel Cancer Screening Program in 2006(11-14). Recent initiatives by cancer advocates and research groups have led to the continuing funding of the Program by the Australian Government in the next five years(15). Although other countries such as Canada, Netherlands, Denmark and Spain do not have a national bowel cancer screening program, various pilots programs and studies are now underway to assess the effectiveness of bowel cancer screening (16).

IFOBT is an accurate test, with studies among the general population reporting a test specificity and sensitivity of at least 80% and 75% respectively(7,17-20). Population screening using iFOBT is also well-accepted by the general population. iFOBT is a simple and user-friendly test, which performs well, compared to other screening strategies such as flexible sigmoidoscopy in the general population. IFOBT screening for colorectal cancer is also cost-effective, with an estimated incremental cost-effectiveness ratio of less than $50,000 per QALY compared to no screening in the general population(13,14,21).

### Screening for colorectal cancer in the CKD population

Unlike screening in people without kidney disease, the costs, benefits, harms of cancer screening in the CKD population is largely unknown. We have conducted a series of systematic reviews and modelled economic evaluations of cancer screening in people with CKD, and found key sources of uncertainty around: patients' preferences and the costs and health outcomes of early cancer detection, the screening test accuracy in CKD, the treatment outcomes and the quality of life of patients with cancer and CKD(13,14,21,22). As such, we have four main reasons for conducting this program of work:

1. The extent of benefits and harms of screening and the screening test characteristics from the general population are not generalisable to individuals with CKD

The performance of the screening test may change when it is applied to different patient subgroups due to the issues of spectrum bias. Higher rates of minute gastrointestinal mucosal bleeding are expected in individuals with CKD because of anti-coagulation use during dialysis, potential uraemic induced platelet defects and the use immunosuppressive agents leading to possible adverse effects on test specificity. Furthermore, previous studies have also shown that the test sensitivity rate increases with the use of low-dose aspirin by increasing the likelihood of bleeding from colorectal neoplasms in the general population(23,24). It is unclear whether low-dose aspirin, or other anti-platelet agents, which are used frequently in the CKD population for the primary and secondary prevention of cardiovascular disease, will have the similar effects on the iFOBT test performance characteristics.

Screening may also be less effective because of higher competing risks of death and lower life expectancy. Many have advocated against routine screening for cancer in patients with end-stage kidney disease (ESKD) because of competing risks of deaths from causes other than cancers(25,26). Some studies have questioned the benefits of screening in people with co-existing co-morbidities and chronic illness such as those with CKD. Previous modelled analyses have shown that people with several chronic conditions such as kidney and lung diseases have a substantially lower gain in life expectancy associated with early stage cancer at diagnosis compared to those without chronic diseases(25,26). However, there is limited information about the types of chronic illnesses and how the aggregation of chronic conditions identifies those who will benefit the most from early cancer detection.

There may also be differences in the potential risk of harms associated with screening in patients with CKD. Identifying trivial and insignificant diseases may lead to unnecessary anxiety; patients may also be treated for disease, which may never be destined to become clinically significant (diagnoses of inconsequential diseases). The screening process may even be more harmful for those with chronic illness such as CKD. For example, subjecting people with reduced kidney function and coexisting cardiac disease to colonoscopic investigations after positive iFOBTs may be detrimental because of the higher risk of cardiovascular events associated with anaesthesia. In addition, previous studies have also reported a higher than expected incidence of bacterial peritonitis among individuals on peritoneal dialysis who have undergone colonoscopic polypectomies(27,28).

*2. There are evidence gaps between guidelines and screening practices for screening colorectal cancer in CKD*.

Our previous work, through systematic reviews and extensive modelled analyses, reports substantial uncertainties regarding the benefits and harms of screening in the context of CKD(22,29,30). Guidelines for screening in patients with reduced kidney function are generally extrapolated from the general population without validated data to support or refute the standard recommended practice. There are also considerable inconsistencies in the recommendations for colorectal cancer screening in CKD patients between countries and regions. The American Transplantation Society recommends annual FOBT screening and flexible sigmoidoscopy screening for all transplant recipients ages 50 years and above(31), whereas the European Best Practice guidelines suggested the use of annual FOBT screening in all transplant recipients above 50 years, in accordance to the national recommendations for the general population(32).

3. There are uncertainties about the cost-effectiveness of screening in CKD

Economic modelling provides an understanding of the potential costs and benefits of colorectal cancer screening and is useful to help inform decisions about implementation of population screening programs. Economic models in the general population have shown that full implementation of the biennial bowel cancer screening program in Australia using iFOBT would reduce bowel cancer mortality and would be an efficient use of limited health resources(13,14). Unlike screening in the general population, analyses of screening colorectal cancer in the transplant population has indicated substantial uncertainties regarding the estimates of the diagnostic test accuracy of iFOBT the transplant population(22). Using the individually-collected costs and clinical estimates from the diagnostic test accuracy study, our trial and modelled economic evaluations will inform clinicians and policy-makers of the direct healthcare costs and effectiveness (health outcomes) of screening compared with no screening in the CKD population.

4. The perspectives and preferences on colorectal cancer screening of the CKD population are unclear

Understanding individuals' perspectives and preferences is necessary to inform and support the development of policies to improve patient engagement and empowerment in decision-making about their own health. Despite the established increased risk of cancer in people with kidney disease, patients with CKD are often understandably preoccupied with the burden of co-existing illnesses such as cardiovascular disease and the complicated dialysis regimens, and may consider the non-imminent issues such as cancer prevention and screening as trivial. Recognising the needs, perspectives and priorities of individual's views and perspectives is paramount to optimise shared decision-making between patients and healthcare providers, and to improve the responsiveness and effectiveness of preventive health care delivery in this at-risk population.

The aims of the DETECT study are to:

1. Determine the test performance characteristics iFOBT screening among individuals with CKD

2. Estimate the incremental costs and health outcomes, and the cost-effectiveness of iFOBT screening compared to no screening in the CKD population

3. Elicit the perspectives and preferences of iFOBT screening among individuals with CKD

## Methods/Designs

### Overview of approach and methods

The DETECT study will utilise both qualitative and quantitative methods to investigate screening colorectal cancer in individuals with CKD. A diagnostic test accuracy study, a cost-effectiveness/cost-utility analysis and qualitative in-depth interviews will be conducted to examine the efficacy, the efficiency and patient perspectives and preferences of colorectal cancer screening in CKD.

#### Study design 1: Diagnostic test accuracy study

A diagnostic test accuracy study will be conducted in the CKD population. The design, the conduct and the reporting of this study is in accordance with the STARD initiatives(33).

##### Patient recruitment and eligibility

Patients ages 35-70, with CKD (stages III-V), CKD-dialysis and CKD-transplant will be recruited from the three main area health services in N.S.W. Although the recommended age for colorectal cancer screening in the general population commences at 50(11), but given the relative increased risk of cancer in the younger transplant population(6) and the greater risk of dying from cardiovascular related causes in the older CKD individuals(34), expansion of the study to include the younger pre-dialysis, dialysis and transplant population is clinically appropriate, and will capture the population that may benefit the most from early cancer detection. Informed consent (verbal and written) will be obtained from all participants.

##### Exclusion criteria

Patients who have a first-degree relative with colorectal cancer or a personal history of colorectal cancer and inflammatory bowel disease, a recent FOBT test (less than one year), who have had a colonoscopy performed within two years, who are medically unfit for a colonoscopy, who are pregnant and who have active gastrointestinal bleeding will be excluded from the study.

##### Baseline data collection

The following information will be collected at baseline. They include: age, gender, self-reported race, co-morbidities, medication use, self and any family history of cancer.

Quality of life (QoL) data will also be collected from all enrolled participants at baselines, after the initial IFOBT screen and after the diagnostic colonoscopy at day 14. We will be using two generic QoL assessment tools: the SF-36 and the Euro-Qol (EQ5D) to capture the QoL associated with screening and the diagnostic procedures.

##### Screening procedure

Eligible participants will be invited to perform the screening tests using the iFOBT kit(35). Two consecutive faecal samples will be required for a single test kit. Test positivity is defined as 100 ng/ml of haemoglobin in either one of the two stool samples

##### Diagnostic procedures

All participants with positive iFOBT screens will be invited to undergo subsequent diagnostic colonoscopies. If polyps are found, polypectomies will be conducted at the time of the diagnostic procedure. If advanced mucosal neoplasia is found, it will be planned for endoscopic mucosal resection (EMR) at a separate procedure. Advanced neoplasia is defined as adenoma with at least one of the following features: 1 cm or more in size, tubulovillous or villous components, or high-grade dysplasia, or any advanced neoplasm 1 cm or more in diameter. EMR is used increasingly frequently for minimally invasive curative resection of benign and early-stage malignant lesions (T1a) throughout the gastrointestinal tract. EMR has the advantage of managing large, sessile polyps in the outpatient setting, which is potentially cost-saving and may improve clinical outcomes in this high risk cohort(36).

All cancers identified will be staged and the participants will be referred to the colorectal surgical and oncology team, depending upon the stage of initial diagnoses. All cancer diagnoses will be reported to the Central Cancer Registry of New South Wales (located within the Cancer Institute of NSW) for all patients with CKD, and the Australia and New Zealand Dialysis and Transplant Registry (ANZDATA) for those on renal replacement therapy (CKD-dialysis and CKD-transplant). The ANZDATA registry is comprehensive database that prospectively collects information on all patients on renal replacement therapy in Australia and New Zealand since 1963. The clinical data includes records of all new cancers except for squamous and basal cell carcinomas. Notification of malignant cancers is a statutory requirement for all health-related institutions in New South Wales. The Central Cancer Registry of New South Wales contains all cancer records and the identifying information for patients diagnosed and treated with cancer within the state of New South Wales since 1972.

##### Reference standard

Clinical follow-up will be the reference standard for all participants. All participants, with or without screen positive results, will be followed clinically two years after their initial screen. To ensure adequate follow-up and accurate calculation of the screening test performance characteristics of cancer, we will compare our records with that of the Central Cancer Registry (CCR) of NSW through data linkage at 2, 5 and 7 years after the initial screens with the attempt to capture all cancer diagnoses.

##### Outcomes

The outcomes of the study will include the following:

1. Prevalence of colorectal cancer and advanced colorectal neoplasia in patients with CKD

2. Screen positivity rate: defined as the proportion of participants with positive screens in the total screened study population

3. Test sensitivity: defined as the number of colorectal cancers and/or advanced neoplasms detected through screening divided by the total number of colorectal cancers and/or advanced neoplasms detected through screening and the total number of cancers and/or advanced neoplasms occurring within the delay in a given period (the follow-up time) after a negative screen.

4. Test specificity: defined as the number of participants with no colorectal cancers and/or advanced neoplasms within the follow-up period divided by the number of participants with no colorectal cancers and/or advanced neoplasms after a negative screen and the number of participants without colorectal cancers and/or advanced neoplasms after a positive screen within the follow-up period.

5. Participation rate of screening among individuals with CKD.

6. Potential harms of screening and the diagnostic colonoscopies, such as bleeding, bowel perforation and the inherent risks of peritonitis, particularly among peritoneal dialysis patients.

7. Direct healthcare costs, including individually- collected screening, diagnostic, treatment and overhead costs.

##### Statistical analyses and sample size calculations

Sensitvity and specificity of iFOBT screening for advanced colorectal neoplasia and cancer will be estimated for (i) CKD (stages 3-5) patients, (ii) dialysis patients, and (iii) transplant patients. For each estimate, the required sample size will be determined by the combined expected prevalence of advanced neoplasia and cancer, the expected sensitivity and specificity, and the required precision of the estimated maximum 90% confidence interval width. For each of the three patient groups, the sensitivity is expected to be 75% and the maximum required 90% confidence interval width is ± 10%. Therefore, 51 cases of advanced neoplasms and cancer will be required. The total sample size and the precision of the estimates of specificity (which is expected to be 90%) for each patient group will be determined by the expected prevalence for that group. In the CKD stages (3-5) group (with a one-year combined estimated prevalence of disease equals to 3.1%), a total of 1637 patients would yield 51 cases and 1586 non-cases. The maximum 90% confidence interval width for specificity is ± 1.3%. Among those on dialysis (with a one-year combined prevalence of disease equals to 3.94%), a total of 1288 patients would yield 51 cases and 1237 non-cases, giving a maximum 90% confidence interval width for specificity of ± 1.4%. Assuming a one-year combined prevalence of disease equals to 4.2% in the transplant population, a total of 1208 patients will again yield a total of 51 cases and 1157 non-cases, giving a maximum 90% confidence interval width for specificity of ± 1.5%.

Across all 3 groups, a total of 4133 participants are required over a 5-year screening period. Assuming a participation rate of 68%, a population size of 6077 CKD patients is required to achieve the target sample size of 4133 for any meaningful analyses.

These sample sizes will provide 80% power to detect a difference of between 3% and 4% in specificity between groups of patients. The small number of expected cases does not allow comparisons in sensitivity to be made between groups.

#### Study design 2: Cost-effectiveness and cost-utility analyses

Using individually-collected clinical estimates such as the prevalence of disease, the test sensitivity and specificity of iFOBT, the screening participation rate, the probability of cancer and adenoma diagnoses, direct costs estimates and utility weights from the diagnostic test accuracy study and from published clinical estimates of randomised controlled trials of screening, trial and modelled -based cost-effectiveness and cost-utility analyses will be conducted to estimates the efficiency of colorectal cancer screening in CKD.

Probabilistic decision analytical models will be developed to estimate the incremental costs and health benefits of screening compared with no screening in the CKD population. Using time-dependent transition probabilities, the models will provide the analytical framework to simulate the natural history of colorectal neoplasms, the screening and the diagnostic process and the outcomes of colorectal neoplasms in individuals with CKD.

##### Outcomes

The outcomes of the study will include the following

1. Healthcare costs of screening and no screening

2. Health outcomes (measured in survival and quality adjusted survival) of screening and no screening

3. Incremental cost-effectiveness ratios as cost per life year saved and cost per quality-adjusted life year gained

#### Study design 3: Qualitative in-depth interviews

This is a qualitative study to investigate the perspectives, experiences, attitudes beliefs and preferences regarding bowel cancer screening in individuals with CKD. Semi-structured face-to-face interviews will be conducted initially with 60 patients (30 participants, 30 non-participants will be sampled from all participating centres). Participants will be purposively selected from the participants and the non-participants of the diagnostic test study (Study 1) to ensure a range of age, ethnicity, co-morbidities and a balance of gender. Data collection will cease when theoretical saturation is reached in the concurrent analysis, which is when little or no new concepts emerge in subsequent interviews. The participants will be asked for their perspectives on: a) knowledge about bowel cancer risk and screening, b) reasons for participating/not participating in the diagnostic screening trial, c) experiences of participating in the screening diagnostic study, d) perceived benefits and harms in participating in screening. All interviews will be audio recorded and transcribed verbatim. The computer software 'HyperRESEARCH 3.0' will be used to assist with storage, coding and searching of qualitative data. Coding and analysis will accord to thematic analysis. The analysis will be performed for all participants collectively; then a sub-analysis will be conducted for screening participants and non-participants to identify and compare differences between both groups.

##### Outcomes

The outcomes of the study will include the following:

1. Identification of barriers and facilitators to participation in bowel cancer screening

2. Understanding of patients decision-making underpinning participation in cancer screening

3. Information to inform policy- and decision-makers about a patient focussed screening program that takes into consideration their needs, priorities and preferences.

##### Ethical considerations

The DETECT study protocol has been approved by the Sydney West Area Health Service Ethics Review Committee (HREC10/WMEAD/13 and SSA/10/WMEAD/54). The University of Sydney, Human Research Ethics committee has also been notified of the approval. The screening test itself is minimally invasive and will not impose any significant harms to the patients. The diagnostic colonoscopies will be performed by experienced gastroenterologists from all participating centres, to prevent any significant complications such as catastrophic bleeding and bowel perforations. The investigator team will monitor all potential electrolytes derrangement resulting from the bowel preparation by collecting and monitoring pre and post-operative serum biochemistry. Information sheets, clearly and succinctly outlining the screening procedure and the associated harms will be given to all eligible patients to read. Patients are in no way obliged to participate and if they do, they can withdraw at any time from the study. They will be informed that their decision to withdraw will not result in any consequences relating to their care provided. Summary of the monthly progress report, outlining any potential complications/harms will be given to all participants and their treating physicians.

## Discussion

The DETECT study is a detailed analysis of the diagnostic test performance characteristics of iFOBT screening, the costs and health benefits of screening in the CKD population and of patient perspectives and preferences regarding colorectal cancer screening. Specifically, this proposed program of research will provide:

1. Estimates of the prevalence for pre-malignant and malignant colorectal neoplasms in the CKD population

2. Estimates of test performance characteristics such as test sensitivity and specificity of iFOBT screening in individuals with CKD

3. Estimates of screening participation rate in people with CKD

4. The incremental costs and health outcomes of iFOBT screening in CKD

5. Patients' perspectives, attitudes, beliefs experiences and preferences regarding screening colorectal cancer

Findings of our research will address the critical issues in chronic disease prevention in Australia and worldwide. This project is uniquely placed to investigate the evidence gaps surrounding the diagnostic test accuracy, the costs and benefits and patients' perspectives and preferences for colorectal cancer screening, with the ultimate objective being to improve the survival and quality of life outcomes in patients with CKD.

## Competing interests

The authors declare that they have no competing interests.

## Authors' contributions

GW, JCC, KH, JRC, AT were responsible for the conceptual design of the study. All authors participated in revisions to the study designs and approved the final study design. GW, JCC, KH were involved in drafting of the manuscript, other authors were involved in the overall revision of the manuscript. All authors are involved in the implementation of the project, and have read and approved the final manuscript.

## Pre-publication history

The pre-publication history for this paper can be accessed here:

http://www.biomedcentral.com/1471-2458/11/516/prepub
